# Cryoballoon ablation for paroxysmal atrial fibrillation mildly improves lung function: An observational study

**DOI:** 10.1097/MD.0000000000035991

**Published:** 2023-11-17

**Authors:** Gaku Oguri, Katsuhito Fujiu, Tsukasa Oshima, Yu Shimizu, Eriko Hasumi, Toshiya Kojima, Issei Komuro

**Affiliations:** a Department of Cardiovascular Medicine, The University of Tokyo, Tokyo, Japan; b Department of Advanced Cardiology, The University of Tokyo, Tokyo, Japan.

**Keywords:** forced expiratory volume, paroxysmal atrial fibrillation, radiofrequency catheter ablation, spirometry, vital capacity

## Abstract

Atrial fibrillation (AF) is the most common arrhythmia and a major public health burden. Catheter ablation (CA) is an effective treatment of AF. Although radiofrequency catheter ablation (RFCA) is the standard practice, cryoballoon ablation (CBA) has become increasingly popular. Pulmonary dysfunction is also associated with AF. As CA targets the pulmonary vasculature, it poses a risk to lung function. However, the effect of CA on respiration in patients with paroxysmal atrial fibrillation (PAF) post-ablation has not yet been assessed. We assessed pulmonary function after CA in a cohort of patients with AF. This prospective, single-center study included 26 patients with symptomatic PAF and 18 patients without PAF. CA techniques include RFCA, CBA, hot balloon ablation, and laser balloon-mediated ablation. Spirometry parameters included vital capacity (VC), forced vital capacity (FVC), forced expiratory volume (FEV1), and peak expiratory flow, which were all measured 6 months post-ablation. AF ablation significantly improved VC (*P* = .04), FVC (*P* = .01), and peak expiratory flow (*P* = .006) in all the patients. In the patients with PAF, we observed a significant increase in FEV1 (*P* = .04). CBA significantly improved VC (*P* = .012) and FVC (*P* = .013). A significant improvement in these pulmonary parameters was achieved, specifically in patients with PAF treated with an ablation protocol with CBA, but not with RFCA or hot balloon ablation. A significant decrease in FEV1 was observed with hot balloon ablation (*P* = .035). Significant improvement in pulmonary parameters was observed specifically in patients with PAF who underwent CBA. CBA may be a more suitable treatment strategy for patients with PAF, particularly those with compromised pulmonary function.

## 1. Introduction

Atrial fibrillation (AF) is the most common sustained cardiac arrhythmia and is associated with an increased risk of stroke and heart failure, resulting in increased patient mortality.^[1]^ As such, AF poses a major public health burden and is recognized as a growing epidemic.^[2]^ Although anti-arrhythmic medications are available, they have limited efficacy and substantial side effects.^[3]^ Therefore, alternative strategies are required to treat AF to improve patient outcomes and reduce the public health burden.

Catheter ablation (CA) is the primary curative therapy for several arrhythmias. CA uses catheters to locate the region of the heart from which arrhythmias are generated, and then damages/ablates them to prevent continued electrical disruption. Radiofrequency catheter ablation (RFCA) is associated with thermal damage and deposition of energy. It is the most commonly used form of CA, with a high success rate of 80% to 90%.^[4]^ Gradual improvements in safety, effectiveness, and efficiency have allowed RFCA to greatly improve the treatment of patients with paroxysmal atrial fibrillation (PAF).^[5]^ Balloon catheters can ablate larger areas of tissue than traditional, single-tip catheters that require point-by-point ablation; hence, balloon-mediated CA techniques such as cryoballoon ablation (CBA) and hot balloon strategies are increasing in popularity. CBA is considered the first-line therapy for patients with PAF and an effective strategy for symptomatic drug-refractory patients. Studies have demonstrated that after the first procedure of CBA, 70% to 80% of patients maintain sinus rhythm. Although the CA procedure poses some risk, its advent has revolutionized the treatment of patients with PAF with an efficacy rate equivalent to ablation by RF.^[6]^ With hot balloon ablation in patients with PAF, a high AF non-recurrence rate was achieved at 24 (93.9%) and 48 weeks (88.8%).^[7]^ A recent study has reported that hot balloon ablation has procedural safety and clinical efficacy comparable to those of CBA.^[8]^ Meta-analyses of trials with long-term and short-term follow-up periods have reported laser balloon ablation to have efficiency and outcome comparable to those of RFA and CA.^[9]^ At the first attempt, a success rate of 89% with recurrence rates of only 12% and 30% for patients with PAF and persistent AF, respectively, at 1 year was achieved.^[10]^

AF is associated with pulmonary dysfunction, and large epidemiological studies have demonstrated that a decline in lung function, determined by forced expiratory volume (FEV1; maximal volume of air exhaled in the first second of forced expiration) and forced vital capacity (FVC; maximal volume of air exhaled), correlates with the incidence of AF.^[11,12]^ Furthermore, patients with chronic obstructive pulmonary disease (COPD)^[13]^ and asthma^[14]^ have a 28% and 1.2-fold increased risk of AF, respectively, and successful management of asthmatic symptoms correlates with reductions in AF.^[15]^ A decline in lung function has also been demonstrated to be predictive of AF.^[11,12]^ Furthermore, pulmonary vein stenosis (PVS) has also been implicated as a potential risk of CA, with a reported prevalence of 3% to 42%, and the overlap of symptoms with common morbidities may account for the variation in the observed frequency.^[16]^ These observations support a clear link between lung function and AF. AF is commonly classified into 5 categories based on episode, duration, and spontaneous termination: first diagnosed, irrespective of its duration or the presence/severity of AF-related symptoms; paroxysmal, marked by short, self-terminating episodes; persistent, recurring episodes requiring intervention to restore sinus rhythm; long-standing persistent, persistent AF with a duration of >12 months; and permanent, when the restoration cannot be achieved by intervention.^[5]^ Thus, the therapy of AF must be customized to the clinical need of individual patients.^[17]^ Although several studies have explored the relationship between lung function and AF, studies on the relationship between the types of AF (PAF and non-PAF) and lung function are scarce.

CA is a standard practice in the treatment of patients with PAF and often targets the pulmonary veins; however, the impact of CA on lung function in patients with PAF has not yet been assessed. Assessment of lung function following CA in patients with PAF would not only indicate whether changes in AF alter respiration in these patients but also determine whether CA produces any adverse effects on the pulmonary system. Therefore, this study aimed to assess pulmonary function following CA in patients with PAF.

## 2. Materials and methods

### 2.1. Study population

This prospective single-center observational study included 44 consecutive patients who underwent initial ablation for symptomatic paroxysmal AF (PAF; n = 26) or non-PAF (n = 18) at the University of Tokyo Hospital between January 2019 and March 2020. Patients aged ≥20 years with only initial AF treatment who provided informed consent and who could undergo respiratory function tests were included. Targeted analyses included patients with pre- and post-treatment data. Patients undergoing dialysis, lung surgery, or treatment for lung cancer, or those with active pneumonia were excluded. All methods were performed in accordance with the relevant guidelines and regulations.^[18]^ Non-PAF cases consisted of patients with persistent AF (defined as episodes persisting for >7 days and not self-terminating) and long-standing persistent AF (continuous AF events extending > 12 months).

CA was prescribed to patients with symptomatic PAF or non-PAF at the physician’s discretion. The procedures were performed under general anesthesia after obtaining written informed consent from all patients. The University of Tokyo Ethics Review Board reviewed and approved the study (approval number: 2650). The study protocol complied with the Declaration of Helsinki and the ethical standards of the review board. This study was not registered as a clinical trial because this is a non-interventional study that only added a respiratory function test to the usual medical examinations.

### 2.2. Ablation protocols

We adopted 4 types of ablation methods for the treatment of patients with PAF according to the indications of the 2017 HRS/EHRA/ECAS/APHRS/SOLAECE Expert Consensus Statement on Catheter and Surgical Ablation of Atrial Fibrillation^[18]^: RFCA, CBA, hot balloon, and laser balloon. Only the RFCA method was used to treat patients without PAF.

A single transseptal puncture was performed after obtaining access to the arteries and veins. Pulmonary vasculature (PV) angiography potential measurements were performed before and after PV isolation using circular mapping catheters. A 3D navigation system (Carto-3; Biosense Webster, Inc.) was used for RFCA, the ablation catheter was Thermocool (Biosense Webster, Inc.), and radiofrequency pulses were delivered around each pulmonary vein point-by-point. During the CA, the output of the RF generator was 35 to 50 W. In the CBA, a 28-mm cryoballoon (ARC-Adv-CB, Arctic Front Advance; Medtronic, Inc., Minneapolis, MN) with an inner lumen mapping catheter (Achieve; Medtronic) was inflated and introduced into each PV orifice through a steerable 15F sheath (FlexCath Advance; Medtronic). Optimal PV occlusion was assessed using contrast injection. Once this was achieved, cryothermal energy was applied to each PV system, first for 180 seconds, and then for 120 seconds. A decapolar catheter was introduced into the superior vena cava, and the phrenic nerve was paced continuously during cryothermal energy application to the right superior and right inferior PVs at a cycle length of 1000 ms, current of 25 mA, and pulse width of 2 ms. Diaphragmatic excursion was assessed by palpation, and diaphragmatic compound motor action potentials were monitored to prevent phrenic nerve injury. Compound motor action potentials were recorded as previously reported.^[19]^ Cryothermal energy application was discontinued when either diaphragmatic excursions were decreased on palpation or a >30% reduction in compound motor action potential amplitude was observed. In the hot balloon method, a hot balloon (SATAKE HotBalloon; Toray Industries, Inc., Tokyo, Japan) with an inner lumen and a J-tip guidewire was inflated in each PV ostium through a 13F deflectable guiding sheath (Treswaltz; Toray Industries). For PV occlusion, the balloon was inflated to 26 to 33 mm in diameter with 10 to 20 mL contrast medium diluted 1:1 with saline. Optimal PV occlusion was assessed by contrast injection, and once this was achieved, a radiofrequency current of 1.8 MHz was applied between the coil electrode inside the balloon and the 4 cutaneous electrode patches on the patient’s back to induce capacitive-type heating of the balloon. A target internal balloon temperature of 70°C was maintained by the delivery of vibratory waves through the lumen into the balloon to agitate the fluid. The radiofrequency-generated thermal energy ranged from 180 to 240 seconds. If a residual PV potential was observed after the first RF application, additional thermal energy was applied for a time equal to that of the first application. We performed phrenic nerve pacing, recorded compound motor action potentials, and monitored them to detect phrenic nerve injury during the application of thermal energy to the right superior PV and right inferior PVs. Esophageal temperature was monitored using an esophageal temperature monitoring system (Esophastar; Japan Lifeline) when hot balloon ablation was applied to the left PVs. To avoid injury when the temperature exceeded 39 °C, cold normal saline was injected into the esophagus via a nasoesophageal tube, after which saline was aspirated from the stomach. All patients received anticoagulant therapy for 4 weeks before ablation and at least 6 months after ablation. Anti-arrhythmic drugs were suspended for 1 week before ablation. The anticoagulant therapy was not interrupted perioperatively. Heparin was administered intraoperatively to maintain an activated clotting time between 300 and 350 seconds.

### 2.3. Respiratory function tests

Respiratory function tests were performed before and 6 months after ablation in all patients using FUDAC-7C (FUKUDA DENSHI Co., Ltd., Tokyo, Japan). All spirometric reference values were determined according to the Japanese Respiratory Society Pulmonary Physiology Committee (JRS 2001).^[19]^ Spirometric parameters included vital capacity (VC; volume of air breathed out after the deepest inhalation), FVC, FEV1, maximal mid-expiratory flow (MMEF), reflecting airflow of large and small airways; peak expiratory flow (PEF; highest forced expiratory flow measured with a peak flow meter); and expiratory reserve volume (ERV; maximal volume of air that can be exhaled from the end-expiratory position).

### 2.4. Statistical methods

Variables are expressed as means ± standard deviations or numbers and percentages, as appropriate. To examine the associations between lung function variables and demographic and clinical parameters, we performed multiple linear regression analyses with age, sex, body mass index, AF type, pulmonary vein isolation (PVI) type, diabetes mellitus, current smoking status, arterial hypertension, and echocardiographic parameters (preoperative data) as dependent variables and each lung function parameter as an independent variable. Continuous variables were analyzed using paired-sample *t* tests. Statistical significance was set at *P* < .05.

## 3. Results

### 3.1. Patient characteristics

The demographic and patient characteristics are listed in Table 1. Of the 44 patients, 26 had PAF and 18 had non-PAF. Age, body mass index, smoking history, hypertension, and preponderance of male participants were similar in both groups. Creatinine levels, left atrial volume index, and CHA2DS2-VASc scores were higher, and EF (%) was lower in the non-PAF group than in the PAF group (Table 1). In the PAF group, ~8% of patients underwent PVI with a laser balloon, ~34% with CBA, ~23% with a hot balloon, and ~34% with RFCA, whereas 100% of patients in the non-PAF group underwent PVI with RFCA (Table 1).

**Table 1:** Patient demographics and clinical characteristics.

	ALL (n = 44)	PAF (n = 26)	Non-PAF (n = 18)	*P* value
Age (yr)	66.1 ± 8	66.5 ± 6.97	65.4 ± 9.3	.65
Sex (F/M)	9/35	7/19	2/16	.27
BMI	25 ± 4	24.9 ± 3.51	25.1 ± 4.5	25 ± 4
AF type (PAF, PeAF, LSAF)	26/14/4			
PVI type (laser balloon, CBA, hot balloon, RFCA)	2/9/6/27	2/9/6/9	0/0/0/18	
Smoker (never, former, current)	17/25/2	11/14/1	6/11/1	.82
Cr (mg/dL)	0.91 ± .21	0.87 ± .19	0.98 ± .21	.094
EF (%)	62.1 ± 10.5	66.03 ± 6.69	56.3 ± 12.2	.0062
LAVI (mL/m^2^)	37.9 ± 17.3	31.34 ± 14.92	47.4 ± 16.1	.0024
HTN (+/-)	29/15	16/10	13/5	.36
DM (+/-)	10/34	5/21	5/13	.45
CHA2DS2-VASc score	1.95 ± 1.31	1.77 ± 1.34	2.22 ± 1.23	.26
Obstractive ventilatory disturbance (%FEV1 < 70)	10/44	6/26	4/18	.94

AF = atrial fibrillation, BMI = body mass index, CBA = cryoballoon ablation, CHA2DS2-VASc = congestive heart failure, hypertension, age, diabetes, previous stroke/transient ischemic attack, sex category- vascular disease, Cr = creatine, DM = diabetes mellitus, EF = ejection fraction, HTN = hypertension, LAVI = left atrial volume index, LSAF = Long-standing persistent AF, PAF = paroxysmal atrial fibrillation, PeAF = persistent AF, RFCA = radiofrequency catheter ablation.

### 3.2. Pulmonary function tests at 6-months post-ablation for AF

**Figure 1.**
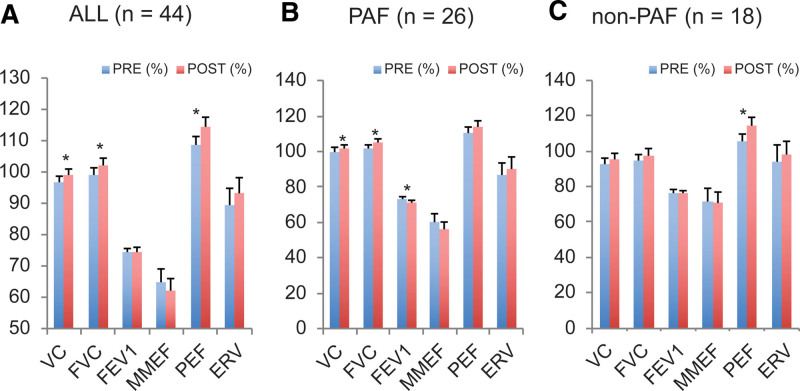
Respiratory function tests in patients with atrial fibrillation. (A) Flow parameters of all patients (n = 44). (B) Flow parameters of patients with PAF (n = 26); and (C) flow parameters of patients without PAF (n = 18). Data are presented as means ± standard deviations. *P < .05 compared to pre-pulmonary vein isolation. PAF = paroxysmal atrial fibrillation.

At 6 months post-ablation, 44 patients with AF were examined to assess respiratory function using the following parameters: VC, FVC, FEV1, MMEF, PEF, and ERV. Based on respiratory function tests, PVI mildly affected pulmonary function. Nevertheless, ablation for AF significantly ameliorated VC (*P* = .04), FVC (*P* = .01), and PEF (*P* = .006) (Fig. 1A). We then separately considered patients with non-PAF and PAF.

In patients with PAF, we observed a significant increase in VC (*P* = .02), FVC (*P* = .002), and FEV1 (*P* = .04) compared with preoperative values (Fig. 1B). In patients without PAF, the increase in PEF at follow-up was statistically significant (*P* = .005) (Fig. 1C). Subsequently, we determined whether different ablation protocols (CBA [n = 9], RFCA [n = 9], and hot balloon therapy [n = 6]) were associated with the observed amelioration of VC and FVC. Only 2 patients underwent laser balloon-mediated ablation, and individual analyses were not performed. Our data revealed a significant improvement in these pulmonary parameters, specifically in patients with PAF who underwent an ablation protocol with CBA, but not with RFCA or hot balloon ablation (Table 2). Hot balloon use led to decreased FEV1 (Table 2). In addition, a logistic regression analysis was performed on the patient background for 4 parameters (VC%, FVC%, PEF%, and FEV%) that demonstrated significant overall change in the cohort. Increases in VC% and FVC% were significantly associated with the absence of diabetes (LR chi2(2) = 4.65, *P* value (Prob > ChiSq) = .0311; LR chi2(2) = 7.68, *P* value (Prob > ChiSq) = .0056, respectively), whereas no significant effectors were observed for increased PEF% and decreased FEV%. However, the small sample size (n = 9) prevented us from achieving robust statistical power.

**Table 2:** Respiratory functions in patients with PAF, according to the Ablation protocol.

	PRE (%)	POST (%)	*P* value
CBA (n = 9)			
VC	102.38 ± 10.13	106.82 ± 10.57	.012
FVC	105.29 ± 9.79	110.14 ± 10.61	.013
FEV1	74.35 ± 2.27	71.81 ± 5.32	.097
MMEF	61.91 ± 10.20	58.21 ± 14.90	.401
PEF	116.74 ± 20.41	123.8 ± 21.69	.11
ERV	101.7 ± 42.50	109.3 ± 40.20	.261
RFCA (n = 9)			
VC	96.61 ± 12.03	96.24 ± 11.10	.761
FVC	98.13 ± 12.26	98.61 ± 11.26	.715
FEV1	75.46 ± 3.77	75.44 ± 6.15	.989
MMEF	67.16 ± 20.41	63.36 ± 23.01	.218
PEF	111.14 ± 15.85	111.35 ± 9.31	.958
ERV	76.04 ± 27.89	77.71 ± 28.76	.831
Hot balloon (n = 6)			
VC	97.55 ± 10.51	101.2 ± 11.76	.186
FVC	100.16 ± 10.82	104.93 ± 12.06	.058
FEV1	71.84 ± 8.44	68.815 ± 9.33	.035
MMEF	56.73 ± 32.51	51.033 ± 29.86	.313
PEF	104.16 ± 15.49	109.23 ± 17.17	.478
ERV	72.76 ± 23.65	80.08 ± 26.16	.275

CBA = cryoballoon ablation, ERV = expiratory reserve volume, FVC = forced vital capacity, FEV1 = forced expiratory volume, MMEF = maximal mid-expiratory flow, PAF = paroxysmal atrial fibrillation, PEF = peak expiratory flow, VC = vital capacity.

## 4. Discussion

Despite the clear link between the decline in lung function and AF, and the potential pulmonary complications of CA, the effect of CA on respiration in patients with PAF has not been previously assessed. The mechanisms underlying the association between compromised lung function and AF are unclear. However, previous studies have suggested the involvement of mechanisms, including hypoxia, systemic inflammation, increased sympathetic nerve activation, and use of COPD treatments, including beta-2 agonists.^[20]^ Among individuals with reduced lung function, hypoxia is common and often results in pulmonary vasoconstriction, leading to pulmonary hypertension and heightened afterload to the right side of the heart.^[21,22]^ Moreover, chronic hypoxia is involved in the regulation of hypoxia-inducible factor 1 and 2 expressions, reactive oxygen species production, vascular inflammation, and blood pressure.^[23]^ Atrial structural remodeling is critical in the development of AF in patients with decreased lung function. In addition, AF progression is related to sympathetic nerve activation, which is often observed in patients with impaired lung function.^[24]^ Use of inhaled beta-2 agonists and anticholinergics, the primary agents used to treat COPD, are also associated with tachyarrhythmia.^[25]^ In this study, we tested lung function before and 6 months after CA in patients with PAF and compared it with lung function in patients with persistent AF (non-PAF). We found that CA led to improvements in lung function at the 6-month follow-up; further improvements were observed in patients with PAF compared with patients without PAF, and CBA had benefits over RFCA and hot balloon CA procedures. These results demonstrate that CA has no adverse effects on lung function in patients with PAF and that some CA procedures may improve respiratory parameters in this disease context.

The analysis of our patient population revealed that approximately 23% of patients (with and without PAF) were diagnosed with obstructive ventilatory disturbance, although none were undergoing treatment. The existing literature states that COPD is present in up to 23% of patients with AF,^[20]^ which supports our findings. Importantly, this observation also suggests that despite the relatively low sample numbers, our patient cohort is representative of the respiratory phenotype present in larger patient populations with AF, and observations made regarding the impact of CA on pulmonary function in this study may have important implications for this population.

PVS was previously considered a prevalent complication with a high rate of 42% (complication of ablation). However, with a better understanding of PVS advancements in ablation procedures, the incidence of severe PVS has been reduced to between 0.32% and 3.4%. In fact, the current Heart Rhythm Society consensus statements do not recommend routine screening for PVS after ablation.^[26]^ When considering patients with and without PAF separately, we observed a significant increase in VC, FVC, and FEV1 compared to preoperative conditions. These data suggest that eliminating the adverse effects of PAF and establishing a simple sinus rhythm are beneficial for improving the pulmonary function. Therefore, lung function was not only conserved following CA but improvements in lung capacity were also observed under some conditions. These findings are in line with those of previous studies in which CA in patients with PAF with chronic lung disease had no adverse effects on heart function compared with patients with normal lung function.^[27]^ The data presented here further support the use of CA in patients with PAF, irrespective of pulmonary function.

We also observed significant improvements in VC, specifically in patients with PAF who underwent an ablation protocol with CBA but not with RFCA or hot balloon ablation. This increase in VC was associated with the absence of diabetes, which is in line with existing literature, as these patients often display a decline in respiratory parameters.^[28]^ The clinical impact of these findings is potentially significant. In general, RFCA delays the progression of AF compared to anti-arrhythmic drug treatment.^[29]^ However, RFCA single-point strategies are insufficient to achieve ablation of a large volume of tissue in a single procedure, and CBA is now the second most frequently used technology after RFCA. In a randomized trial, CBA was superior to RFCA in terms of efficacy for the treatment of patients with drug-refractory PAF, and no significant difference regarding safety was observed.^[30]^ Moreover, CBA has a significantly lower incidence of pericardial effusions/cardiac tamponade than RFCA.^[31]^ Collectively, these data support the use of CBA over other CA techniques, particularly in patients with reduced respiration.

Despite the respiratory improvements observed with CBA, hot balloon CA caused a decrease in postoperative FEV1 (%). This phenotype can often be attributed to the obstruction of air escaping from the lungs, and reduced FEV1 (%) has been suggested to be associated with chronic AF in large population studies; thus, such reductions in FEV1 within subsets of our patient cohort are not surprising.^[32]^ Wakamatsu et al^[8]^ have reported differences in lesion formation in patients who underwent CBA and those who underwent the hot balloon procedure, which may account for the differences in functional outcomes observed in our study. However, increased patient numbers and monitoring of lesion formation following CA are necessary to elucidate the cause of functional differences between the CBA and hot balloon CA patient cohorts.

This study had several limitations. First performing a comprehensive evaluation of pulmonary vein occlusion would have been useful if the residual air volume could be tested; however, we were unable to perform this evaluation because of the limited duration of each test. Second, the follow-up evaluation was performed 6 months postoperatively, and no long-term evaluation was completed; therefore, the respiratory results of the chronic phase remain unknown. Third, some of the respiratory function results (particularly ERV and MMEF) were provided by the equipment in numbers and not as percentages, making it difficult to extend the validity and medical significance of the data across patients with inter-individual variability. Fourth, we did not assess AF recurrence as an outcome of ablation. Finally, the sample size was small, which limits the statistical significance of the findings.

## 5. Conclusions

Pulmonary function is not compromised 6 months after CA in patients with PAF, and patients undergoing CBA experience a significant improvement in lung capacity. CBA may be a more appropriate solution for PAF than RFCA, particularly in patients with compromised lung function. Our observations justify the need for long-term studies with larger patient cohorts to confirm these conclusions and translate the findings into improved clinical management for patients with PAF.

## Acknowledgments

Editorial support in the form of medical writing, assembling tables, creating high-resolution images based on the authors’ detailed directions, collating author comments, copyediting, fact checking, and referencing was provided by Editage and Cactus Communications.

## Author contributions

**Conceptualization:** Gaku Oguri.

**Data curation:** Gaku Oguri.

**Formal analysis:** Gaku Oguri.

**Funding acquisition:** Gaku Oguri.

**Methodology:** Gaku Oguri, Toshiya Kojima.

**Project administration:** Gaku Oguri.

**Resources:** Tsukasa Oshima, Toshiya Kojima.

**Software:** Gaku Oguri.

**Supervision:** Katsuhito Fujiu, Issei Komuro.

**Validation:** Eriko Hasumi, Toshiya Kojima.

**Visualization:** Gaku Oguri.

**Writing – original draft:** Gaku Oguri.

**Writing – review & editing:** Gaku Oguri, Katsuhito Fujiu, Tsukasa Oshima, Yu Shimizu, Eriko Hasumi, Issei Komuro.
